# On the determination of *χ*^(2)^ in thin films: a comparison of one-beam second-harmonic generation measurement methodologies

**DOI:** 10.1038/srep44581

**Published:** 2017-03-20

**Authors:** Artur Hermans, Clemens Kieninger, Kalle Koskinen, Andreas Wickberg, Eduardo Solano, Jolien Dendooven, Martti Kauranen, Stéphane Clemmen, Martin Wegener, Christian Koos, Roel Baets

**Affiliations:** 1 Photonics Research Group, Department of Information Technology (INTEC), Ghent University (UGent) - imec, Technologiepark-Zwijnaarde 15, 9052 Ghent, Belgium; 2 Center for Nano- and Biophotonics (NB-Photonics), Ghent University, Technologiepark-Zwijnaarde 15, 9052 Ghent, Belgium; 3 Institute for Microstructure Technology (IMT), Karlsruhe Institute of Technology (KIT), Hermann-von-Helmholtz-Platz 1, 76344 Eggenstein-Leopoldshafen, Germany; 4 Institute of Photonics and Quantum Electronics (IPQ), Karlsruhe Institute of Technology (KIT), Engesserstraße 5, 76131 Karlsruhe, Germany; 5 Optics Laboratory, Tampere University of Technology, P.O. Box 692, 33101 Tampere, Finland; 6 Institute of Applied Physics, Karlsruhe Institute of Technology (KIT), Wolfgang-Gaede-Straße 1, 76128 Karlsruhe, Germany; 7 Department of Solid State Sciences, Faculty of Sciences, Ghent University, Krijgslaan 281/S1, 9000 Ghent, Belgium; 8 Institute of Nanotechnology, Karlsruhe Institute of Technology (KIT), Hermann-von-Helmholtz-Platz 1, 76021 Karlsruhe, Germany

## Abstract

The determination of the second-order susceptibility (*χ*^(2)^) of thin film samples can be a delicate matter since well-established *χ*^(2)^ measurement methodologies such as the Maker fringe technique are best suited for nonlinear materials with large thicknesses typically ranging from tens of microns to several millimeters. Here we compare two different second-harmonic generation setups and the corresponding measurement methodologies that are especially advantageous for thin film *χ*^(2)^ characterization. This exercise allows for cross-checking the *χ*^(2)^ obtained for identical samples and identifying the main sources of error for the respective techniques. The development of photonic integrated circuits makes nonlinear thin films of particular interest, since they can be processed into long waveguides to create efficient nonlinear devices. The investigated samples are ABC-type nanolaminates, which were reported recently by two different research groups. However, the subsequent analysis can be useful for all researchers active in the field of thin film *χ*^(2)^ characterization.

Second-order nonlinear optical processes enable a whole range of applications used in a variety of areas, ranging from research oriented tools to widespread commercially available devices^1^. One major field of application are the light sources based on second-order nonlinear effects, such as frequency doubled lasers^2^ (e.g. most green laser pointers), optical parametric oscillators (OPO’s, sold by plenty of laser manufacturers^3^,^4^), THz generators^5^ and quantum light sources^6^,^7^. Another application domain is optical signal processing. Electro-optic modulators^8^, widely used in fiber-optic communication, and optical correlators^9^,^10^ are two prominent examples of such signal processing devices. Also various characterization methods utilized in research are based on second-order nonlinear processes, like second-harmonic imaging microscopy^11^,^12^ and surface second-harmonic generation to probe material interfaces^13^,^14^.

Exploiting the advancements made in integrated optics, the aforementioned devices can be implemented in photonic integrated circuits (PIC’s) to decrease cost and footprint, increase the device efficiency and add functionality to existing PIC’s. In integrated optics, materials that are deposited or grown in thin films are of vital importance, as they very often serve as a starting point in the fabrication of waveguide circuits through lithography techniques^15^. The integration of nonlinear functions and the search for second-order nonlinear thin film materials are both active fields of research. This makes the existence of easy and reliable methods for the characterization of the second-order nonlinearity in thin films undoubtedly an asset.

The strength of any second-order nonlinear process is characterized by the second-order susceptibility tensor *χ*^(2)^. As a tensor of rank 3, *χ*^(2)^ consists of 3^3^ = 27 elements. Typically, the number of independent, non-zero elements is much lower because of symmetry properties^16^. Nonetheless, determining the *χ*^(2)^ tensor elements of a nonlinear material remains a delicate task.

There are several different approaches to determine *χ*^(2)^ of a material. Often, electro-optic modulation via the Pockels effect is exploited for that purpose^17^,^18^. However, this technique requires electrodes and typically uses waveguiding structures, which means additional design and fabrication effort is needed. A more straightforward way to determine *χ*^(2)^ is to exploit the effect of second-harmonic generation (SHG). In this well-known second-order nonlinear process, light at the fundamental frequency *ω* interacts with a nonlinear material to generate light at the double frequency 2*ω*. SHG in waveguiding structures can be very efficient if the phase matching condition is satisfied and the modal overlap is large^19^. But as these requirements demand a substantial amount of engineering, the use of waveguiding structures is not very suitable for material characterization. Hence simple free-space transmission experiments are usually preferred.

Shortly after the first demonstration of second-harmonic generation^20^, a standard technique, called the Maker fringe method, was developed to determine *χ*^(2)^ of nonlinear crystals^21^. Over the years various alterations of the formalism have been suggested to include effects such as anisotropy, absorption and reflections in multilayer media^22^,^23^,^24^,^25^. The technique is based on Maker’s finding that the pattern of the transmitted second-harmonic (SH) power generated in a bulk nonlinear crystal reveals the so-called Maker fringes when the crystal is rotated^26^. These fringes occur since the SHG process is not phase matched and the SH waves generated at different locations in the crystal can interfere constructively or destructively depending on the effective length of the crystal that is changed by the rotation. The Maker fringes serve as characteristic features that allow for a reliable extraction of *χ*^(2)^ when the experimental data is fitted with theoretical expressions. The downside of this approach is that distinct Maker fringes are only visible if the thickness of the nonlinear material is significantly larger than the coherent build-up length^16^. This is not the case for submicron thin films which have attracted attention in recent years due to their application perspectives in integrated optics^27^,^28^,^29^,^30^,^31^. Therefore, there is a demand for alternative measurement methodologies that are better suited for thin film *χ*^(2)^ characterization.

In parallel to the early research on bulk effects in nonlinear crystals, the field of surface second-harmonic generation emerged. In media with inversion symmetry second-order effects are forbidden in the electric dipole approximation, i.e. *χ*^(2)^ = 0. However, at the interface between two centrosymmetric media, the inversion symmetry is naturally broken and second-order effects are allowed again^13^,^16^. The effect was first reported in calcite by Terhune and co-workers^32^. Since then, it has been used as a research tool for investigating the interface structure of various centrosymmetric materials, for studying the adsorption of molecular monolayers and monitoring surface chemistry^33^,^34^,^35^,^36^,^37^. In a typical experiment the strength of the transmitted or reflected SHG is measured as a function of the angle of incidence and/or the polarization state^38^. This allows determination of the surface *χ*^(2)^ tensor elements, which on its turn provides information about the atomic structure of the interface, the concentration and orientation of adsorbed molecules, etc. As in surface SHG the *χ*^(2)^ layer is only a few atoms thick, no Maker fringes are observed.

In this work we use elements from both the worlds of SHG in bulk media and surface SHG to determine *χ*^(2)^ of ultrathin films deposited on a centrosymmetric substrate. More specifically, the samples characterized are second-order nonlinear ABC-type nanolaminate optical metamaterials on glass substrates. These materials were recently introduced to the community by two independent research groups at Karlsruhe Institute of Technology (KIT)^39^ and Ghent University (UGent)^40^. The UGent and KIT group utilize different *χ*^(2)^ measurement techniques. In the present work, the two measurement methodologies with their corresponding calibration methods and theoretical models will be analyzed and the main sources of error will be identified. Both groups use a one-beam second-harmonic generation characterization technique. It has been shown that accurate determination of *χ*^(2)^ for thin films is possible with a sophisticated two-beam geometry, that is, an arrangement with two fundamental beams incident on the sample^41^. However, the alignment of the two fundamental beams is challenging and can introduce additional errors.

The approach of the UGent group is similar to the Maker fringe technique where for a fixed polarization of the fundamental beam the SH power is recorded while rotating the sample. Also in the UGent measurements fringes appear and they are used in the data fitting to obtain *χ*^(2)^. However, the source of these fringes is not the same as in a traditional Maker fringe experiment. It is exploited that the SH signal generated in the thin film interferes with SH radiation generated at the glass-air interface (backside of the sample). For very thin films that do not have a very high nonlinearity (films in this work are thinner than 100 nm and have *χ*^(2)^ ~ 1 pm/V) a fringe pattern shows up in the recorded SH powers. To implement the glass-air surface nonlinearity in the UGent model, a formalism from the field of surface SHG is utilized. Because a femtosecond laser is used in the measurements, also temporal walk-off effects occurring in the substrate need to be taken into account for a correct *χ*^(2)^ characterization. The KIT group uses a different approach where the angle of incidence is fixed and the s- and p-polarized SH power is recorded as a function of the polarization of the incident fundamental beam. An adapted form of a standard Maker fringe model is used to accommodate for the varying polarization state rather than a change in angle of incidence.

In summary, we present a comparison between two measurement techniques with their corresponding calibration methods and theoretical models for determination of the *χ*^(2)^ tensor elements of ABC-type metamaterials. For both techniques, the major advantages but also the shortcomings and most important sources of error will be identified. In order to compare the reliability of the absolute values of the determined *χ*^(2)^ tensor elements for the two different techniques, both research teams exchanged their nanolaminates. That is, identical samples are measured by both teams with their respective measurement technique and the results are compared to identify potential sources of uncertainty. Note that these findings are not restricted to ABC-type nanolaminate metamaterials but hold for thin nonlinear films in general. Therefore this paper can be used as a guideline for researchers wanting to characterize their own *χ*^(2)^ thin films.

## Materials and Setups

The ABC-type nanolaminates analyzed in this article consist of very thin layers (on the order of 1 nm) of three amorphous (and thus centrosymmetric) materials A, B and C that are deposited alternately through atomic layer deposition (ALD). At each of the interfaces the inversion symmetry is broken locally and consequently second-harmonic (SH) waves can be generated. The layers are combined to an ABCABC… stack that also breaks the inversion symmetry globally. In this way destructive interference between the generated SH waves is avoided, as would happen in an ABAB… stack. The nanolaminate of Ghent University consists of the three materials TiO_2_, Al_2_O_3_ and In_2_O_3_. It is deposited on a 500 μm BOROFLOAT^®^ 33 substrate and has a total thickness of 66 nm obtained from ellipsometry and transmission electron microscopy. The nanolaminate of Karlsruhe Institute of Technology is composed of the three materials Al_2_O_3_, HfO_2_ and TiO_2_. It has a thickness of 75 nm obtained from ellipsometry and the substrate is borosilicate glass of the first hydrolytic class with a thickness of 170 μm. More information about the fabrication of these ABC-type thin films can be found in the Methods section. In the remainder of this paper the nanolaminates of UGent and KIT will be referred to as ITA (In_2_O_3_/TiO_2_/Al_2_O_3_) and HTA (HfO_2_/TiO_2_/Al_2_O_3_) respectively.

**Figure 1:**
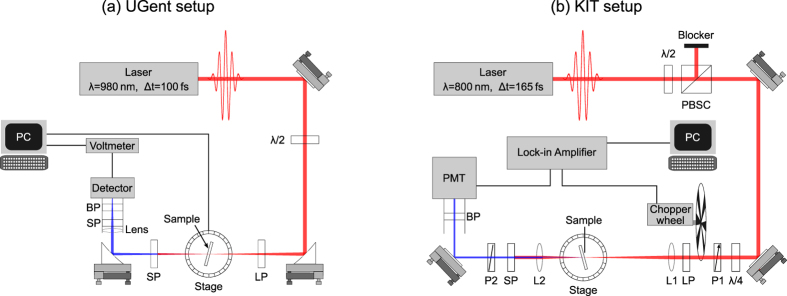
Schematic representation of the experimental setups at (**a**) UGent and (**b**) KIT for characterizing the second-order nonlinearity of the nanolaminates by means of second-harmonic generation (SHG). PBSC: polarizing beam-splitter cube, P1, P2: wire-grid polarizers, *λ*/2, *λ*/4: half- and quarter-wave plate, LP: longpass filter, L1, L2: lenses, SP: shortpass filter, BP: bandpass filter, PMT: photomultiplier tube.

Due to the limited thickness of these nanolaminates, the generated SH power is quite small, which means a powerful laser combined with sufficiently sensitive detection is necessary for generating and detecting the SH power. The UGent and KIT setups are shown in Fig. 1(a,b), respectively. In the UGent setup the light source is a commercial Ti:Sapphire laser (Mai Tai HP from Spectra-Physics) emitting 100 fs pulses at a wavelength of 980 nm, a repetition rate of 80 MHz and an average power close to 1 W. A half-wave plate is used to rotate the polarization direction of the linearly polarized laser light to p-polarization. Two parabolic mirrors of 5 cm focal length are used to focus the laser beam on the sample and to collimate it again after passing through the sample. The focusing brings the beam diameter (1/*e*^2^) down from 1.2 mm to 52 μm. This leaves us with a depth of focus, i.e. 2 × Rayleigh length, of 4.5 mm, giving enough tolerance for the alignment of the sample in the focal plane. Surrounding the sample there is a long- and shortpass filter. The former filters out any spurious light at wavelengths below 800 nm, while the latter suppresses the laser light at the fundamental wavelength and lets the SH light through. It is important to place these filters right before and after the sample to eliminate parasitic SHG originating in the beam path. The sample is placed on a motorized rotation stage (Thorlabs CR1-Z7) and allows for measurement automation. A lens is placed in front of the femtowatt detector (Thorlabs PDF10A) which compensates for beam displacements induced by the rotation of the sample. There is also a bandpass filter that removes part of the stray light. Moreover it allows to check that the detected light is truly SHG: by tuning the fundamental wavelength until the SH wavelength falls out of the passband of the filter and checking that the detected signal vanishes. During a measurement the sample is rotated and the SH power is measured as a function of the incidence angle.

In the KIT setup, the nonlinear samples are excited using laser pulses from a Ti:Sapphire mode-locked oscillator (*Tsunami* from Spectra-Physics) operating at a repetition rate of 80.5 MHz, a pulse duration of 165 fs FWHM (full width at half maximum), a centre wavelength of 800 nm and an average power of around 2 W. The excitation power can be set by rotating a half-wave plate which is followed by a polarizing beam-splitter cube. A quarter-wave plate is used to get a circularly polarized beam. Subsequently, a wire-grid polarizer sets the beam at a constant power to a linear polarization at an angle tunable between 0° (s-polarized) and 90° (p-polarized). The laser beam is chopped to allow for lock-in detection and passes a longpass filter that removes potential parasitic SHG prior to the sample. The fundamental pump beam is then loosely focused onto the sample to a spot diameter (1/*e*^2^) of about 50 μm using a lens with a focal length of 200 mm. The diverging light is collimated by a lens with a focal length of 100 mm and filtered by a shortpass (cut-off wavelength 700 nm) and a bandpass filter (centre wavelength 400 nm, spectral width 40 nm). Finally, the SH power is detected using a Hamamatsu R4332 photomultiplier tube. To distinguish between s- and p-polarized SH light, a wire-grid polarizer is placed in the beam path between the sample and the photomultiplier tube. The sample is mounted on a rotation stage which allows to set the angle of incidence. In addition, the sample mount features two goniometers that can be used to correct for tilt of the sample relative to the fundamental beam and relative to the rotation axis of the rotation stage.

## Models and calibration

**Figure 2:**
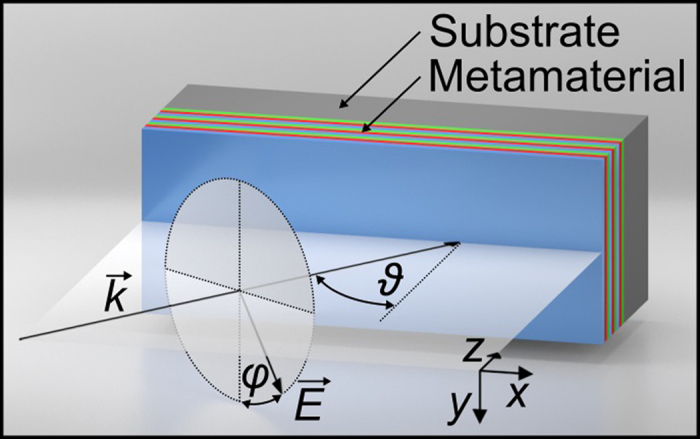
Illustration of the excitation geometry including the angle of incidence *ϑ* and the polarization angle *φ*. Incident s-polarization corresponds to *φ* = 0°, p-polarization to *φ* = 90°. Reprinted from ref. 39, with the permission of AIP Publishing.

The individual nanoscale A, B and C layers of the nanolaminate are not considered in the analysis, instead an effective medium approach is used. Since these ABC-type materials show a *C*_∞*v*_ symmetry with respect to the film normal, the non-vanishing second-order susceptibility tensor components for SHG are 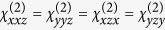, 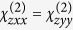 and 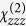, where *z* is the film normal and *x* and *y* are two orthogonal in-plane directions, see Fig. 2 36. We assume that these tensor elements are real as we are working at wavelengths far from resonance. Note that the presented theoretical models are readily applicable to thin nonlinear films that posses the same symmetry properties, e.g., ZnO thin films^29^. After adapting the non-zero tensor elements, the models can be used for films with a different symmetry as well.

In general the SH electric field components generated in an achiral thin film with in-plane isotropy (*C*_∞*v*_ symmetry) can be expressed as 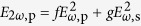 and *E*_2*ω*,s_ = *hE*_*ω*,s_*E*_*ω*,p_, where p and s stand for the p- and s-polarized components and *E*_*ω*_ refers to the electric field amplitude of the incident fundamental beam^42^. The quantities *f, g* and *h* are coefficients that depend on the nonlinear susceptibility tensor components, the incidence angle, the linear optical properties, the frequency and the thickness.

In the UGent model, two contributions to the total SH signal are considered: SH waves generated at the front interface of the sample, where the thin film is deposited, and SH waves generated at the back glass-air interface. Furthermore, the thin film is assumed to have zero thickness. This translates into a physical model where polarized sheets are located at the front and back interface of the sample. The nonlinearities are described by surface second-order susceptibility tensors 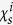 for the air-thin film (*i* = ABC) and the glass-air interface (*i* = glass)^38^. In our definition of 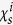 we use internal electric fields, meaning that the nonlinearities are defined with respect to the electric fields inside the thin film (for the front interface) and inside the glass (for the back interface). The surface susceptibility of the thin film is converted to a bulk susceptibility by dividing 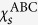 by the thin film thickness. We neglect multiple reflections (inside thin film and glass substrate) and we also do not take into account the birefringent character of the thin films. Instead we use an average refractive index. Note that the degree of anisotropy is only a few percent for the ITA sample^40^ and negligible for the HTA sample according to ellipsometry measurements. To justify these simplifications, we compared our model to a more advanced model taking into account reflections, anisotropy and finite film thickness, see Supplementary Fig. S4, showing the limited impact of these factors. When including these effects the involved equations become cumbersome and less straightforward to interpret physically. Intuitively, one can understand that the zero film thickness is a good approximation for films much thinner than the wavelength and the coherence length. As we are dealing with small refractive index contrasts (*n*_glass_ ≈ 1.5 and *n*_ABC_ ≈ 2), reflections will also be small as long as the incidence angle is not too large. For p-polarized light, the reflection only rises significantly once Brewster’s angle is passed. Our analysis shows that we can safely neglect reflections if we keep the incidence angle below 70°. Since the incident fundamental beam is p-polarized in the UGent setup, the generated SH waves will also be p-polarized. For a monochromatic p-polarized plane wave at the fundamental frequency with electric field amplitude *E*_*ω*,in_ incident on the sample, we can find the following expression for the transmitted electric field at the SH frequency^43^,^44^


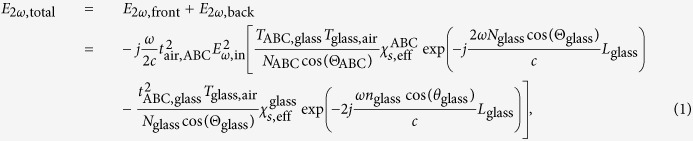


with 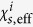 the effective surface second-order susceptibility defined as


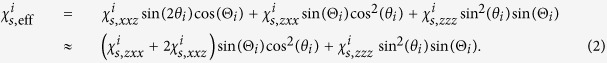


In these relations, parameters specified at the fundamental frequency *ω* (SH frequency 2*ω*) are written in lower case letters (capital letters). The quantities *T*_*i,j*_ and *t*_*i,j*_ are the Fresnel transmission coefficients (for p-polarized light) propagating from medium *i* to *j, N*_*i*_ and *n*_*i*_ are the refractive indices of medium *i*, Θ_*i*_ and *θ*_*i*_ are the propagation angles with respect to the surface normal *z, L*_glass_ is the thickness of the glass substrate and *c* is the speed of light. The refractive indices of the samples are: *n*_ABC_ = 1.9556, *N*_ABC_ = 2.0975, *n*_glass_ = 1.4633 and *N*_glass_ = 1.4766 for the ITA sample, and *n*_ABC_ = 1.901, *N*_ABC_ = 1.996, *n*_glass_ = 1.513 and *N*_glass_ = 1.5297 for the HTA sample. The refractive indices for the nanolaminates are determined through ellipsometry; for the glass they are found in the datasheet supplied by the manufacturer. The approximation in Eq. 2 can be done because *θ*_*i*_ ≈ Θ_*i*_ for samples with low dispersion. This also means that 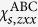 and 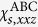 cannot be determined separately in the UGent measurement. Instead we use the combined value 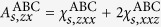 as a fitting parameter. Using the non-approximated version of Eq. 2 in the regression analyis of the data leads to diverging results. In principle, 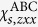 and 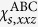 can be determined separately even for low-dispersion materials by studying the polarization signature of carefully chosen polarization combinations for the input and/or output beams. For example, if an s-polarized fundamental beam is used only 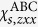 will be probed. But in practice it is difficult to obtain reliable information from this type of measurements in the UGent setup, as the SH powers are close to the detection limit for the considered samples.

As we rotate the sample and detect the SH power, the front and back contributions will interfere resulting in an angle-dependent fringe pattern. These interference fringes form the basis of the UGent calibration method and can be used for *χ*^(2)^ extraction. A reference measurement needs to be done on a blank glass substrate to determine its surface second-order susceptibility 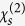. For BOROFLOAT^®^ 33, this measurement and the obtained 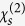 values are shown in Supplementary Fig. S1. For several other types of glass, the surface nonlinear susceptibility can be found in ref. 45. The average detected power is given by *P*_2*ω*_ = *K*_1_|*E*_2*ω*,total_|^2^, where *K*_1_ is a function of the pulse duration, the repetition rate of the laser, the spot size and the transmission of the optics. As it can be difficult to know all these properties accurately, it is often preferred to determine *K*_1_ from a calibration measurement. In the UGent calibration method we use the known nonlinear susceptibility of the back glass-air interface, to fit *K*_1_ and the unknown susceptibility tensor elements of the thin film simultaneously. However, this calibration method cannot be used for films with a very strong nonlinear response, since the interference fringes will no longer be discernible.

Equation 1 is only strictly valid for monochromatic waves. When working with short laser pulses, there will be a temporal walk-off between the SH pulse generated at the front and back surface of the sample which Eq. 1 does not account for. The pulse at the fundamental wavelength will travel at a different group velocity through the substrate than the SH pulse generated at the front and thus there will be a small delay between both generated SH pulses. This reduces the depth of the interference fringes. If this effect is not taken into account, it will result in an overestimation of *χ*^(2)^ of the thin film. The temporal walk-off effect is implemented in the model by introducing sech^2^ pulses^46^


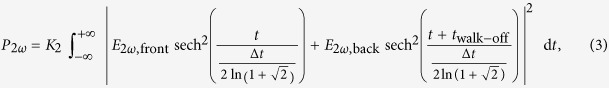


with Δ*t* the FWHM pulse duration, *t*_walk−off_ the walk-off time and *K*_2_ again a calibration constant. The walk-off time increases for increasing incidence angles and can be expressed as 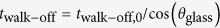, with *t*_walk−off,0_ the walk-off time for normal incidence.

The KIT model is based on the approach of Herman and Hayden^23^ which solves the nonlinear wave equation and satisfies the boundary conditions for the SH wave at all interfaces of the sample: at the air-film, the film-substrate, and the substrate-air interface. The approach has been generalized for arbitrary polarization of the fundamental pump beam^39^. The model takes into account the thickness of the nanolaminate and therefore also the phase mismatch between fundamental and SH wave which becomes of importance for thick or very dispersive nonlinear films. However, the effect of multiple reflections inside the nanolaminate as well as inside the glass substrate at both the fundamental and SH frequency are neglected due to the small index difference. In addition, the small birefringence of the nanolaminate is neglected. Also, the formalism does not take into account the SH wave that is generated by the second-order nonlinearity of the substrate-air interface. For the p/s-polarized peak SH power which is generated in the sample the following expression is derived


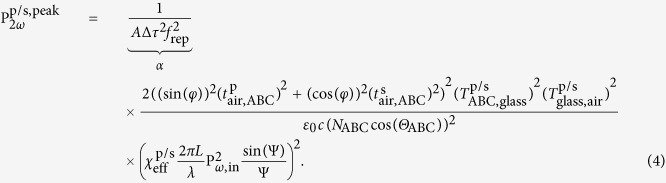


Parameters specified at the fundamental frequency *ω* (SH frequency 2*ω*) are written in lower (upper) case letters. 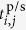 and 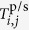 are the Fresnel transmission coefficients (for p/s-polarized light) propagating from medium *i* to *j*, and *n*_*i*_ and *N*_*i*_ are the refractive indices of medium *i*. The constant *c* is the vacuum speed of light, *A* is the spot size of the laser at the focus, Δ*τ* is the FWHM of the temporal pulse and *f*_rep_ is the repetition rate of the laser. The quantity P_*ω*,in_ corresponds to the average incident laser power outside the nonlinear film, *L* is the thickness of the nonlinear film, *λ* is the vacuum wavelength of the fundamental beam and *φ* is the pump polarization angle in air with *φ* = 0°, 90° for s- and p-polarization, respectively. Possible phase mismatch between fundamental and SH waves is accounted for by the quantity 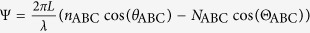, where *θ*_ABC_ and Θ_ABC_ are given by Snell’s law 
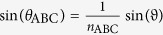
 and 
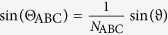
 with the angle of incidence *ϑ*. The quantity *α* is independently determined by a reference measurement. This is advantageous because of the potentially inaccurately determined spot size *A* and pulse width Δ*τ* of the fundamental beam. The parameter 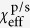 is the effective second-order susceptibility for p/s-polarized SHG, which takes the following form for *C*_∞*v*_ symmetry


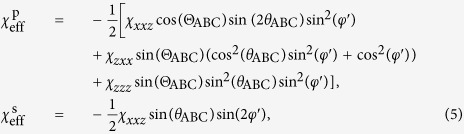


In this relation, the quantity *φ*′ is the polarization angle of the pump inside the nonlinear material which is defined by 
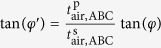
. The excitation geometry is depicted in Fig. 2 where also *φ* and *ϑ* are defined. The refractive indices of the samples are: *n*_ABC_ = 1.979, *N*_ABC_ = 2.1935, *n*_glass_ = 1.4661 and *N*_glass_ = 1.4839 for the ITA sample, and *n*_ABC_ = 1.9242, *N*_ABC_ = 2.0194, *n*_glass_ = 1.5163 and *N*_glass_ = 1.5405 for the HTA sample.

To determine the second-order nonlinearity of a sample the s- and p-polarized SH power is recorded as a function of the polarization of the fundamental beam *φ* for a fixed angle of incidence. The detected s- and p-polarized SH power are fitted simultaneously with the theoretical expressions presented in Eq. 4, where the three independent tensor elements of *χ*^(2)^ are the corresponding fitting parameters. The parameter *α* is determined in an independent Maker fringe measurement of a Y-cut quartz crystal with known second-order nonlinearity of 
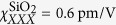
47. This is done immediately after the *χ*^(2)^ measurement on the nanolaminate in order to avoid the influence of possible laser fluctuations.

## Results and Discussion

In this section the weaknesses and strengths of the two measurement techniques will be discussed and the most important sources of errors for the deduction of *χ*^(2)^ will be identified. Since the measurement methodologies are different, the relevant sources of errors will be also different for the KIT and UGent approach. The fitting of the measurement data in the following section is carried out using a nonlinear least-squares algorithm. The nonlinear regression is done assuming a constant error, i.e. equal weights for all data points.

### UGent measurements and fitting

**Figure 3:**
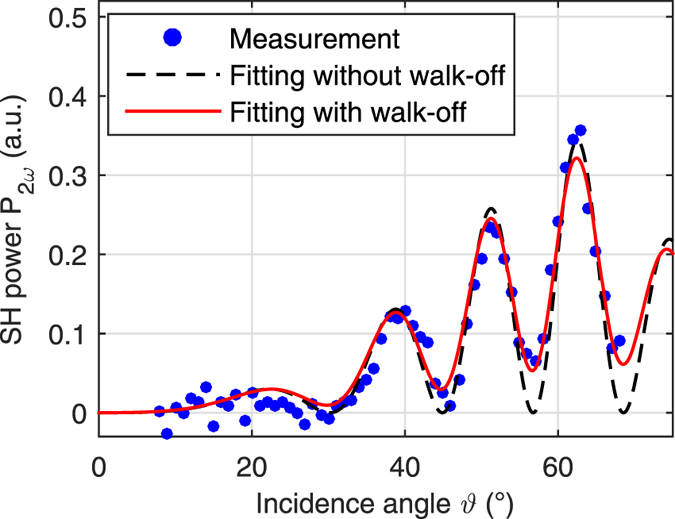
Fitting the measurement data for a blank BOROFLOAT^®^ 33 substrate without temporal walk-off (black dashed line) and with walk-off (red full line). Negative SH powers for the measurement data are obtained due to subtraction of background noise. The regression analysis gives us a walk-off of 45.3 fs and a substrate thickness of 484.97 μm. The thickness agrees well with the specified thickness of (500 ± 20) μm.

The reference measurement to determine the nonlinear surface susceptibility of a blank glass substrate was performed at Tampere University of Technology using a picosecond laser, see Supplementary Fig. S1. From this reference measurement, which was calibrated against a Y-cut quartz crystal, we get: 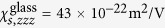 and 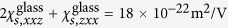. To find the walk-off time *t*_walk−off,0_, the measurement of a blank substrate needs to be repeated with the UGent setup, see Fig. 3. The data is fitted using the model with and without walk-off (see Equations 1 and 3, except the ABC-type coating on the front surface needs to be replaced with glass). The fitting parameters are the substrate thickness *L*_glass_, the constants *K*_1_ and *K*_2_ and the walk-off time *t*_walk−off,0_. The fitting of the substrate thickness is necessary to ensure that the extrema of the fringes in the fitted curve are positioned at the right incidence angles. Incidence angles greater than 70° are not considered, since the theoretical model does not hold any more due to the unaccounted reflections. Supplementary Fig. S3 illustrates the consequences for the fitting when larger incidence angles are included. In Fig. 3 we see that the visibility of the fringes is lower for the measurement data than it is for the fitted curve when walk−off is not included in the model. Including the temporal walk−off effect clearly improves the agreement between the measurement and the fitted curve. The regression analysis gives a walk-off time of *t*_walk−off,0_ = 45.3 fs. This result will be used later in the fitting of the data of the ABC-type thin films. From the refractive index versus wavelength graph in the datasheet^48^ we get 37 fs as a very rough estimate (estimating 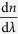 graphically), which agrees well with the fitted value.

**Figure 4:**
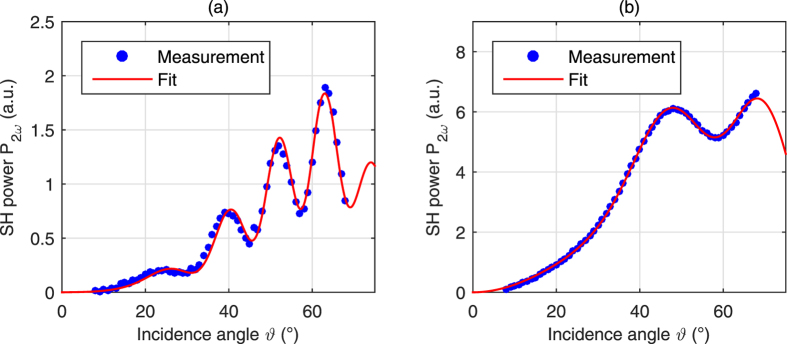
Measured and fitted SH power vs incidence angle for (**a**) ITA sample and (**b**) HTA sample in the UGent setup (laser wavelength of 980 nm). As the substrate is thinner for the HTA sample, there are fewer interference fringes.

The measured SH powers and corresponding fitted curves for both the ITA and HTA sample are shown in Fig. 4. The theoretical model is described by Eq. 3, where *K*_2_, *L*_glass_, 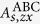 and 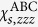 are the fitting parameters. The fitting results for the ITA sample are: 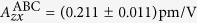, 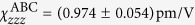 and *L*_glass_ = (498.26 ± 0.16) μm. For the HTA sample we get: 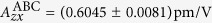, 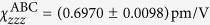 and *L*_glass_ = (170.310 ± 0.040) μm. The quoted uncertainties are the standard deviations following from nonlinear regression analysis; these only reflect the uncertainty due to statistical intensity fluctuations. The standard deviations are kept low by averaging over many read-outs for a single angle of incidence.

There is also an uncertainty on the incidence angle which can impact the final results. Since we are working with an motorized rotation stage, the relative angle setting is very precise (repeatability < 1′ according to specifications). The main uncertainty originates in the setting of the reference angle: after we have placed the sample, we need to manually set the angle corresponding to normal incidence. We estimate that this results in a maximum systematic error of 2°. Table 1 illustrates how this systematic error will influence the retrieved *χ*^(2)^ values. The uncertainties are several times larger than the standard deviations following from the nonlinear regression, and currently they are the main source of uncertainty in the UGent measurements.

Another aspect that needs to be stressed is the importance of proper alignment in this type of measurements. The depth of focus is only 4.5 mm, so care must be taken to avoid the sample from moving out of the laser focus while rotating it. We achieve this by aligning at two widely spaced incidence angles: we maximize the signal for both angles by iteratively adjusting the mirrors in our setup. When the rotation axis of the sample is not positioned in the laser focus, it will cause an apparent shift in the weights of the fitted nonlinear tensor elements, i.e. certain elements will become smaller, others larger. From simulations we estimated that displacements of the rotation axis with respect to the focus on the order of 1 mm can cause the tensor elements to change by several 10’s of percent, which would make the induced error of similar magnitude to the error due to uncertainty on the incidence angle. To avoid possible alignment issues one can choose to focus the laser beam less tightly, but again this requires a more sensitive detection mechanism.

The reproducibility of the measurements was tested by doing multiple measurements on the same sample. In between measurements the sample was removed from the sample holder and put back into place. The setup was also intentionally misaligned and realigned. Including error boundaries, these measurements gave identical results.

### KIT measurements and fitting

An advantage of the KIT approach is the simplicity in sample alignment. Since *ϑ* is constant during a measurement there is no risk of rotating the sample out of focus. Moreover, the influence of the slightly inhomogeneous sensitivity of the active region of the PMT is eliminated since the SH light spot on the PMT does not move during the measurement.

**Figure 5:**
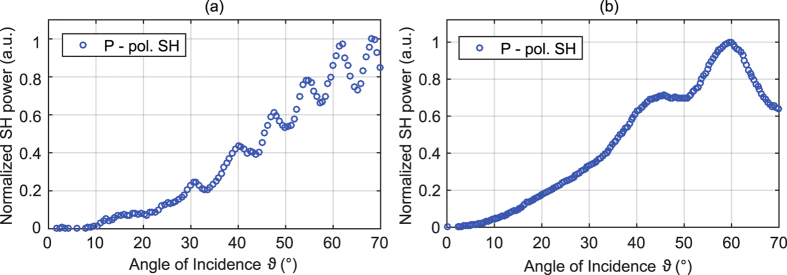
p-polarized SH power as a function of the angle of incidence *ϑ* for p-polarized fundamental beam obtained from (**a**) the ITA sample and (**b**) the HTA sample. These measurements are done using the KIT setup (laser wavelength of 800 nm). Both measurements show oscillations in the SH power which can be attributed to interference effects between SH waves generated in the nanolaminate and SH waves generated at the back surface of the glass substrate.

The most important source of error for the KIT technique is the fact that the KIT model does not take into account interference effects between SH wave emanated from the nanolaminate and the glass back surface. In fact, these interference fringes are also visible in the KIT setup as depicted in Fig. 5 where the p-polarized SH power is plotted as a function of the angle of incidence *ϑ* for a p-polarized fundamental beam for the HTA and ITA sample. Note that in the KIT setup, a fundamental wavelength of 800 nm instead of 980 nm in the UGent setup is used. This is why the interference patterns are different from the ones measured with the UGent setup. In order to estimate the error that is introduced by neglecting the influence of interference, several measurements at different angles of incidence are performed for both the HTA and ITA sample. These angles are chosen such that constructive interference (*ϑ*_constr._) or destructive interference (*ϑ*_destr._) occurs. For these two extreme cases the determined value for *χ*^(2)^ will be overestimated or underestimated, respectively. An additional measurement at an intermediate angle (*ϑ*_inter._) between those two specified angles will give a good indicator of the true value of *χ*^(2)^.

**Figure 6:**
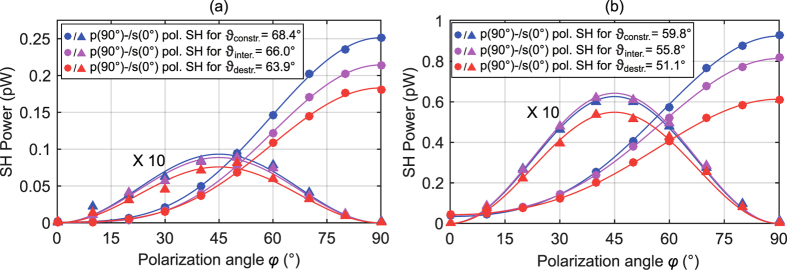
s- (triangles) and p-polarized SH power (circles) and corresponding fitting curves (lines) as a function of the polarization angle *φ* for an average excitation power of P_*ω*,in_ = 200 mW obtained from (**a**) the ITA sample at 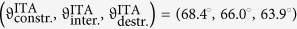 and (**b**) the HTA sample at 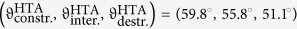. Here, *φ* = 0°, 90° corresponds to incident s- and p-polarization, respectively. To quantify the impact of interference we perform three measurements at different angles of incidence where *ϑ*_constr._ and *ϑ*_destr._ denote the angles of incidence under which constructive and destructive interference occurs, respectively and *ϑ*_inter._ denotes an intermediate angle of incidence. Note that data and fit of the s-polarized SH power is multiplied by a factor 10 for better visibility.

Figure 6 shows three separate measurements of the s- (triangles) and p-polarized (circles) SH power and the corresponding fitted theoretical expressions (lines) as a function of *φ* obtained for the ITA and the HTA sample. The measurements are performed for three different angles of incidence where the blue, magenta and red markers and lines correspond respectively to 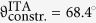, 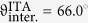 and 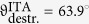 for the ITA sample and 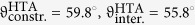 and 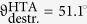 for the HTA sample. For better visibility of the s-polarized SH power the data sets and corresponding fitting curves are multiplied by a factor of 10. The determined values of the *χ*^(2)^ tensor components depend significantly on the angle of incidence *ϑ*, as summarized in Table 2 where the last column contains the average value of the determined tensor elements and the corresponding standard deviation. Note that for all deduced tensor elements the error following from curve fitting is well below 10%. It is interesting to observe that for the HTA sample the decrease of the determined value of 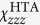 is very pronounced when going from constructive to destructive interference. On the other hand, the determined off-diagonal tensor elements vary only little for the three different angles. This finding suggests that the off-diagonal tensor elements of the HTA nanolaminate are much larger than the off-diagonal tensor elements of the glass surface, which would result in *ϑ*-independent values of these tensor components. Indeed, only minor interference occurs for s-polarized SH power when the fundamental beam polarization is defined by *φ* = 45°, in this case only 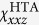 is addressed, see Supplementary Fig. S5(b). Furthermore, no interference effects are visible for p-polarized SH power when the fundamental beam is s-polarized, in this case only 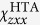 is addressed, see Supplementary Fig. S6. For the ITA sample the deduced value for 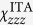 decreases more moderately when changing the angle of incidence from *ϑ*_constr._ to *ϑ*_destr._. Additionally, also the calculated value of the off-diagonal tensor element 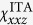 decreases with decreasing *ϑ*. In fact, s-polarized SH power for a fundamental beam polarization of *φ* = 45° exhibits major interference effects which can explain this decrease in 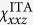, see Supplementary Fig. S5(a). The large variation of 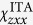 is attributed to the fact that the low level of p-polarized generated SH power for s-polarized fundamental beam is close to the detection limit. Note that the visibility of the fringes and therefore this source of error is eliminated for thicker or more strongly nonlinear films as it was shown for 290 nm thick, strongly nonlinear ZnO/Al_2_O_3_ nanolaminates^49^.

As an additional source of error, an imprecisely calibrated angle of incidence is investigated. The angle calibration is done by studying the back reflected fundamental beam close to normal incidence and trying to make it overlap with the incident beam by rotating the sample. In order to illustrate the effect on the deduction of *χ*^(2)^, an error of Δ*ϑ* = ±1.0° will be assumed. Note that using the same technique in the UGent setup gives larger errors, as a focusing element with a smaller focal length is used. The effect is quantified by performing *χ*^(2)^ measurements on the ITA and HTA sample at angles of incidence of 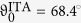 and 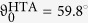 (blue data in Fig. 6) while the data is evaluated at angles of incidence of *ϑ*_0_ and *ϑ*_0_ ± Δ*ϑ*. The fitting results for Δ*ϑ* = ±1.0° are *χ*_*zzz*_ = (0.86 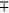 0.05) pm/V, *χ*_*xxz*_ = (0.264 ± 0.004) pm/V and *χ*_*zxx*_ = (0.216 ± 0.004) pm/V for the HTA sample and *χ*_*zzz*_ = (1.25 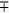 0.02) pm/V, *χ*_*xxz*_ = (0.169 ± 0.008) pm/V and *χ*_*zxx*_ = (0.063 ± 0.003) pm/V for the ITA sample. It can be seen that the values for the tensor elements change by roughly 5%. This can be understood as an upper error bound since the error in the angle of incidence of Δ*ϑ* = ±1.0° is chosen pessimistically.

The reproducibility of the measurements was also checked by doing multiple measurements on the same sample. The setup was deliberately misaligned and realigned before each measurement. The results of these measurements were in very good agreement with one another.

Lastly, the Maker fringe reference measurement on a Y-cut quartz plate that is necessary for the determination of the quantity *α* in Eq. 4 is briefly discussed. To validate the stability of the calibration measurement and the corresponding fit, a set of Maker fringe measurements is performed, see Supplementary Fig. S2. The fitting results vary by less than 1% for consecutive measurements. Therefore it can be assumed that the calibration technique does not introduce any significant error.

## Conclusions

In conclusion, two different thin film *χ*^(2)^ measurement methodologies, their corresponding calibration techniques and underlying theoretical models are described and the most important sources of errors are identified. The techniques are tested with two similar ABC-type nanolaminates grown by ALD on glass substrates, fabricated by two independent groups from UGent and KIT.

The UGent methodology takes into account the interference from the backside of the substrate and it even exploits this for calibration purposes. During a measurement, the sample needs to be rotated, and thus care must be taken when aligning the sample such that it stays in the laser focus. Currently, the main source of error in the UGent method is related to the setup rather than the theoretical model, namely the systematic error on the incidence angle. The obtained *χ*^(2)^ values and associated overall error margins are 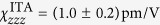 and 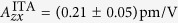 for the ITA sample. For the HTA sample we have 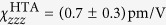 and 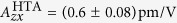. These overall error margins include the error listed in Table 1 and the error introduced by curve fitting. By improving the sensitivity of the current setup, the off-diagonal tensor elements could be identified separately. This could also allow to focus the beam more loosely, which eases the alignment and leads to a reduced error on the angle.

The KIT technique for *χ*^(2)^ determination is very robust against misalignment between the sample and the fundamental beam, since the sample is not moved during the measurement. Additionally, the high detection sensitivity allows to determine the three tensor components individually. The main source of error is due to the fact that the KIT formalism used for the evaluation does not take into account interference effects of the SH waves generated in the nonlinear film and at the glass back surface. The deduced values and corresponding overall error bounds for the tensor elements are 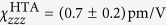, 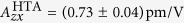 and 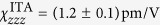, 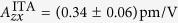 for the HTA and the ITA sample, respectively. The specified error bounds include the error due to interference effects, the errors introduced by curve fitting and the errors due to an imprecise setting of the angle of incidence. The interference effect, the main source of error, is reduced significantly if thicker samples are examined or if samples with higher nonlinearity are introduced.

**Figure 7:**
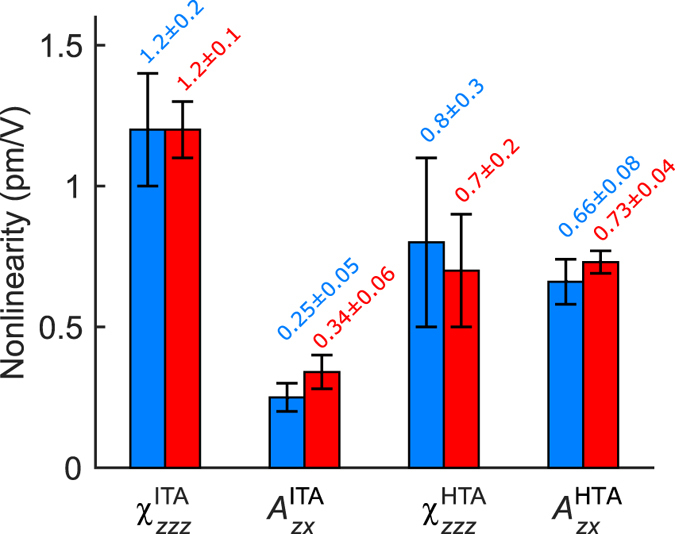
Overview of the obtained *χ*^(2)^ tensor elements for the 2 samples under investigation via the UGent measurement (blue) and the KIT measurement (red). The tensor elements determined in the UGent measurement are scaled according to Miller’s rule in order to fairly compare the values at a reference wavelength of 800 nm.

Finally, we compare the *χ*^(2)^ values determined with the two different techniques. Since, the two presented setups use lasers at different wavelengths, we compensate for the dispersion of *χ*^(2)^ by considering Miller’s rule^50^. As a reference wavelength we choose the fundamental wavelength used in the KIT setup, i.e. 800 nm. Therefore we need to scale the tensor elements determined in the UGent setup by using the relation


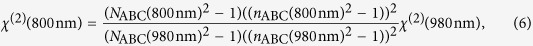


where *χ*^(2)^ can be *χ*_*zzz*_ or *A*_*zx*_. The *χ*^(2)^ values at the reference wavelength of 800 nm are summarized graphically in Fig. 7. The values for the relevant tensor element *χ*_*zzz*_ determined with the different techniques match very well for both the ITA and HTA sample and also for the off-diagonal elements the error bounds overlap. It is also interesting to compare these values with previously reported values^39^,^40^. For the ITA sample, ref. 40 reported a value of 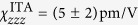, compared to a value of 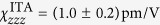 presented in this paper. On the one hand, this is caused by the use of a larger reference surface nonlinearity for the glass substrate in ref. 40 (the surface nonlinearity of BK7 calibrated against 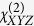 of quartz was used). Additionally, the thin film thickness was underestimated. Indeed, we now have measurements performed via electron microscopy and ellipsometry that give a thickness of 66 nm while the previous estimate of 50 nm was done solely by summing up the ALD calibrated thickness of each layer (0.7 nm × 24 × 3 = 50 nm). The remaining discrepancy can be explained by all the factors discussed above with a dominant impact of the temporal walk-off. The HTA sample reported here is nominally identical to the ACB sample with inverted order of growth and adapted thicknesses of the individual layers reported in the main text of ref. 39 for which a dominant *χ*^(2)^ tensor element of *χ*_*zzz*_ = 0.43 pm/V can be estimated. This is in fair agreement with the value of 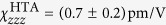 reported here. The discrepancy is due to the fact that the effect of SHG from the back surface was not accounted for in ref. 39. Additionally, in ref. 39 the *χ*^(2)^ deduction was done without the reference measurement on quartz which led to an underestimation of *χ*^(2)^.

In summary, both *χ*^(2)^ measurement techniques represent attractive alternatives to the traditional Maker fringe technique if thin film samples are investigated. For thin films generating high levels of SH power with negligible interference from the substrate, the KIT technique is very well suited. On the other hand, the UGent technique is the technique of choice when very thin and weakly nonlinear films are investigated, provided that a reliable measurement of the backside nonlinearity is available. Because of the interesting application perspectives of nonlinear thin films in photonic integrated circuits, the use of reliable *χ*^(2)^ measurement techniques is of considerable importance to the research community.

## Methods

### UGent sample fabrication

The UGent ABC-type thin film is deposited through plasma-enhanced atomic layer deposition. The materials A, B and C are TiO_2_, Al_2_O_3_ and In_2_O_3_. After cleaning the glass substrate in O_2_ plasma, the deposition is done by alternating 10 s pulses of the corresponding metal-organic precursor at a pressure of 6.0 × 10^−5^ bar and O_2_ plasma pulses at 1.2 × 10^−5^ bar. The substrate temperature is 120 °C throughout the full deposition process. The plasma is generated at an RF power of 200 W and a frequency of 13.56 MHz. The precursors used for Ti, Al and In are tetrakis(dimethylamino)titanium 99% (Strem Chemicals P.Nr. 93-2240), trimethyaluminium 97% (Strem Chemicals P.Nr. 93-1360) and tris(2,2,6,6-tetramethyl-3,5-heptanedionato)indium 99% (Strem Chemicals P.Nr. 49-2200). The number of cycles for each TiO_2_, Al_2_O_3_ and In_2_O_3_ layer are 12, 7 and 70. This gives individual layer thicknesses of about 0.7 nm. The ABC period is repeated 24 times which gives a total of 24 × (12 + 7 + 70) = 2136 ALD cycles.

### KIT sample fabrication

The substrates for the KIT sample are borosilicate glass of the first hydrolytic class with a thickness of 170 μm for SHG measurements and silicon wafers for ellipsometry measurements. Before deposition, the substrates are cleaned with acetone and dry-blown by N_2_. In order to limit deposition on the substrate front side, the back surface is covered with high temperature resistant masking tape. The film is fabricated by ALD using a Savannah 100 system by Cambridge Nanotech at a deposition temperature of 150 °C. The precursors for Al, Hf and Ti and O are trimethyaluminium 97% (Sigma-Aldrich P.Nr. 257222), tetrakis(dimethylamido)hafnium(IV) ≥99.99% (Sigma-Aldrich P.Nr. 455199), titanium(IV) isopropoxide 99.999% (Sigma-Aldrich P.Nr. 377996) and hydrogen peroxide 30% (Merck P.Nr. 107209), respectively. The reaction chamber is constantly flushed with 20 sccm of Ar, unless differently specified. Further parameters are given in Supplementary Table S1. The sample consists of 32 ABC macrocycles. The numbers of growth cycles for the individual layers in each macrocycle are 8, 8 and 12 for Al_2_O_3_, HfO_2_ and TiO_2_ corresponding to estimated layer thicknesses of 0.9 nm, 0.9 nm and 0.3 nm, respectively.

## Additional Information

**How to cite this article:** Hermans, A. *et al*. On the determination of χ^(2)^ in thin films: acomparison of one-beam second-harmonicgeneration measurement methodologies. *Sci. Rep.* **7**, 44581; doi: 10.1038/srep44581 (2017).

**Publisher's note:** Springer Nature remains neutral with regard to jurisdictional claims in published maps and institutional affiliations.

## Supplementary Material

Supplementary Information

## Figures and Tables

**Table 1:** Error margins on the bulk susceptibilities and substrate thicknesses as a consequence of the systematic error on the incidence angle (±2°) for the UGent measurement technique.

	ITA sample	HTA sample
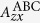 (pm/V)	0.21 ± 0.04	0.60 ± 0.07
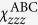 (pm/V)	1.0 ± 0.2	0.7 ± 0.3
*L*_glass_ (μm)	498 ± 5	170 ± 1

Results are shown both for the ITA and HTA sample.

**Table 2:** Deduced *χ*^(2)^ tensor elements for different angles of incidence *ϑ*_constr._, *ϑ*_destr._ and *ϑ*_inter._ corresponding to constructive interference, destructive interference and an intermediate case respectively. Results are shown for the HTA and ITA sample.

HTA sample	*ϑ*_constr._ = 59.8°	*ϑ*_inter._ = 55.8°	*ϑ*_destr._ = 51.1°	Average
*χ*_*zzz*_ (pm/V)	0.862	0.685	0.450	0.667 ± 0.207
*χ*_*xxz*_ (pm/V)	0.264	0.262	0.236	0.254 ± 0.016
*χ*_*zxx*_ (pm/V)	0.216	0.231	0.227	0.225 ± 0.008
**ITA sample**	***ϑ***_**constr.**_** = 68.4°**	***ϑ***_**inter.**_** = 66.0°**	***ϑ***_**destr.**_** = 63.9°**	**Average**
*χ*_*zzz*_ (pm/V)	1.252	1.155	1.088	1.165 ± 0.082
*χ*_*xxz*_ (pm/V)	0.168	0.144	0.132	0.148 ± 0.018
*χ*_*zxx*_ (pm/V)	0.063	0.030	0.049	0.047 ± 0.017

Because the interference from the substrate is not accounted for, different results are obtained for different angles of incidence. The last column shows the average values and the corresponding standard deviations for each tensor component.
