# Supplementary material for: On the determination of χ(2) in thin films: a comparison of one-beam second-harmonic generation measurement methodologies
Source: Sci Rep. 2017 Mar 20;7:44581. doi: 10.1038/srep44581 (PMC5357890; doi:10.1038/srep44581)
Supplement: Supplementary Information [file srep44581-s1.pdf]

On the determination of $\chi^{(2)}$ in thin films: a comparison of one-beam second-harmonic generation measurement methodologies (Supplementary Information)

Artur Hermans[1,2,*,†], Clemens Kieninger[3,4,†], Kalle Koskinen[5], Andreas Wickberg[6], Eduardo Solano[7], Jolien Dendooven[7], Martti Kauranen[5], Stéphane Clemmen[1,2], Martin Wegener[6,8], Christian Koos[3,4,*], and Roel Baets[1,2]

[1]Photonics Research Group, Department of Information Technology (INTEC), Ghent University (UGent) - imec, Technologiepark-Zwijnaarde 15, 9052 Ghent, Belgium
[2]Center for Nano- and Biophotonics (NB-Photonics), Ghent University, Technologiepark-Zwijnaarde 15, 9052 Ghent, Belgium
[3]Institute for Microstructure Technology (IMT), Karlsruhe Institute of Technology (KIT), Hermann-von-Helmholtz-Platz 1, 76344 Eggenstein-Leopoldshafen, Germany
[4]Institute of Photonics and Quantum Electronics (IPQ), Karlsruhe Institute of Technology (KIT), Engesserstraße 5, 76131 Karlsruhe, Germany
[5]Optics Laboratory, Tampere University of Technology, P.O. Box 692, 33101 Tampere, Finland
[6]Institute of Applied Physics, Karlsruhe Institute of Technology (KIT), Wolfgang-Gaede-Straße 1, 76128 Karlsruhe, Germany
[7]Department of Solid State Sciences, Faculty of Sciences, Ghent University, Krijgslaan 281/S1, 9000 Ghent, Belgium
[8]Institute of Nanotechnology, Karlsruhe Institute of Technology (KIT), Hermann-von-Helmholtz-Platz 1, 76021 Karlsruhe, Germany
[*]artur.hermans@ugent.be; christian.koos@kit.edu
[†]These authors contributed equally to this work

Supplementary Figure S1: Reference measurement Tampere University of Technology

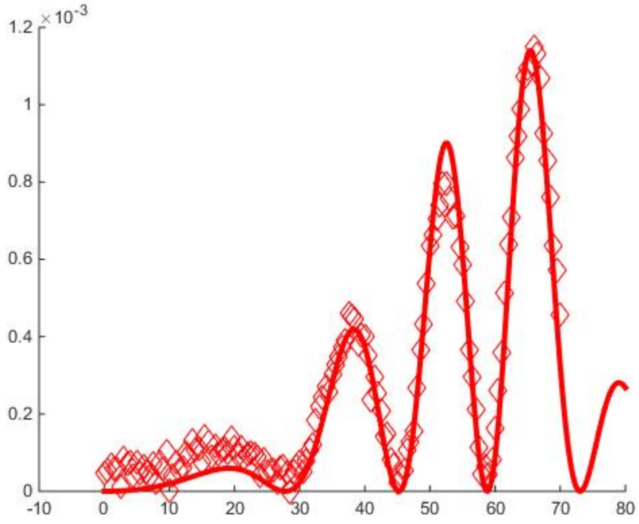

Reference measurement for a blank BOROFLOAT® 33 substrate (500 µm thickness) conducted at the Optics Laboratory, Tampere University of Technology. The horizontal axis represents the incidence angle ϑ (in degrees) and the vertical axis shows the SH power $P_{2\omega}$ (in arbitrary units). Both the incident fundamental wave and the generated SH wave are p-polarized. The fitted $\chi_s^{(2)}$ values are $\chi_{s,zzz} = 43 \times 10^{-22}\,\mathrm{m}^2/\mathrm{V}$ and $2\chi_{s,xxz} + \chi_{s,zxx} = 18 \times 10^{-22}\,\mathrm{m}^2/\mathrm{V}$ when referenced against quartz with known second-order nonlinearity $\chi_{XXX}^{\mathrm{SiO_2}} = 0.6\,\frac{\mathrm{pm}}{\mathrm{V}}$.

Supplementary Figure S2: Reference measurement on Y-cut quartz plate

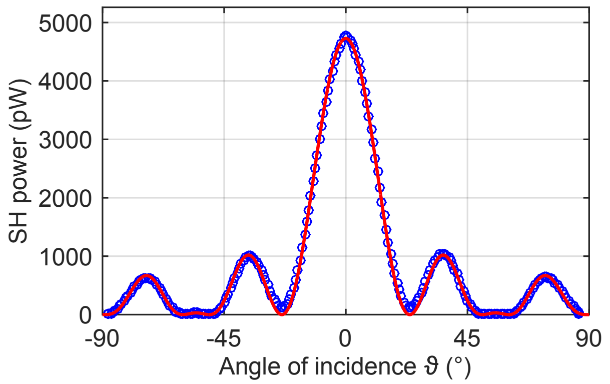

Typical Maker fringe calibration measurement and fit of a Y-cut quartz plate. The p-polarized SH power is recorded as a function of the angle of incidence ϑ for p-polarized fundamental beam with an excitation power $\mathrm{P}_{\omega,\mathrm{in}} = 100\,\mathrm{mW}$. The theoretical model for the fit is also given by Eq. 4 in the main text. However, it has to be noted that the quartz plate is not supported upon a substrate. This can be accounted for by replacing the refractive index of the substrate by the refractive index of air. The effective susceptibility for the Y-cut quartz crystal reads

$$\chi_{\mathrm{eff}}^{\mathrm{p}} = \chi_{XXX}^{\mathrm{SiO_2}} \left(\cos(\Theta_{\mathrm{SiO_2}}) \left(\cos^2(\theta_{\mathrm{SiO_2}}) - \sin^2(\theta_{\mathrm{SiO_2}})\right) - 2\sin(\Theta_{\mathrm{SiO_2}}) \sin(\theta_{\mathrm{SiO_2}}) \cos(\theta_{\mathrm{SiO_2}})\right), \quad (1)$$

with $\chi_{XXX}^{\mathrm{SiO_2}} = 0.6\,\frac{\mathrm{pm}}{\mathrm{V}}$ [1]. There are two free fitting parameters. The first one is the thickness L of the quartz plate which determines the position of the individual Maker fringes. Note that fitting the thickness is a quite common approach in the evaluation

of Maker fringe experiments[2]. The second fitting parameter is $\alpha = \frac{1}{A\Delta\tau^2 f_{\mathrm{rep}}^2}$ that is needed for the evaluation of Eq. 4 in the main text. The fitting results are $L = (119.486 \pm 0.007)\,\mu\mathrm{m}$ and $\alpha = (2.340 \pm 0.007)\,10^{18}\,1/\mathrm{m}^2$. For three consecutive measurements on different spots of the quartz plate the following mean values and corresponding standard deviations are obtained $L = (119.24 \pm 0.22)\,\mu\mathrm{m}$ and $\alpha = (2.338 \pm 0.015)\,10^{18}\,1/\mathrm{m}^2$. Since the standard deviations of the fitting results for an individual measurement, as well as for consecutive measurements are negligible it can be assumed that the calibration measurement itself introduces insignificant error on the $\chi^{(2)}$ tensor elements of the nanolaminates. Note that due to the symmetry of the data relative to $\vartheta = 0°$ it can be excluded that the angle of incidence on the sample was calibrated incorrectly. Additionally, due to the good agreement between data and fit in magnitude and position of the individual Maker fringes it can be assumed that the quartz plate is not rotated out of focus during the measurement.

Supplementary Figure S3: Comparison of different theoretical models for the glass substrate measurements

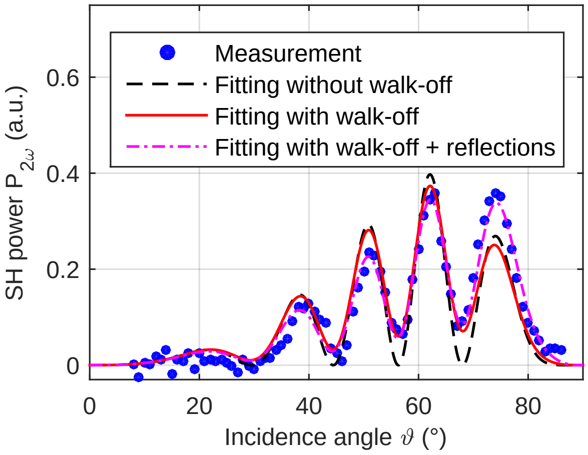

Measurement and fitting for a blank BOROFLOAT® 33 substrate with known $\chi_s^{(2)}$. For incidence angles larger than 70° there is a clear discrepancy between the fitted curve and the measurement when reflections are not included. When reflections are included a good fit is also obtained for the larger angles. We only include single reflections at the glass-air interfaces, not multiple reflections within the substrate (so no Fabry-Pérot type effects). See Ref. 3, 4 for more details.

The model without reflections is described by (see also main text)

$$
\begin{aligned}
E_{2\omega,\mathrm{total}} &= E_{2\omega,\mathrm{front}} + E_{2\omega,\mathrm{back}} \\
&= -j\frac{\omega}{2cN_{\mathrm{glass}}\cos\left(\Theta_{\mathrm{glass}}\right)} t^2_{\mathrm{air,glass}} T_{\mathrm{glass,air}} E^2_{\omega,\mathrm{in}} \\
&\left(\chi^{\mathrm{glass}}_{s,xxz}\sin\left(2\theta_{\mathrm{glass}}\right)\cos\left(\Theta_{\mathrm{glass}}\right) + \chi^{\mathrm{glass}}_{s,zxx}\sin\left(\Theta_{\mathrm{glass}}\right)\cos^2\left(\theta_{\mathrm{glass}}\right) + \chi^{\mathrm{glass}}_{s,zzz}\sin^2\left(\theta_{\mathrm{glass}}\right)\sin\left(\Theta_{\mathrm{glass}}\right)\right) \\
&\left[\exp\left(-j\frac{2\omega N_{\mathrm{glass}}\cos\left(\Theta_{\mathrm{glass}}\right)}{c}L_{\mathrm{glass}}\right) - \exp\left(-2j\frac{\omega n_{\mathrm{glass}}\cos\left(\theta_{\mathrm{glass}}\right)}{c}L_{\mathrm{glass}}\right)\right].
\end{aligned} \tag{2}
$$

The notations are the same as in the main text.

The model with single reflections is described by

$$
\begin{aligned}
E_{2\omega,\text{total}} &= E_{2\omega,\text{front}} + E_{2\omega,\text{back}} \\
&= -j\frac{\omega}{2cN_{\text{glass}}\cos\left(\Theta_{\text{glass}}\right)} t^2_{\text{air,glass}} T_{\text{glass,air}} E^2_{\omega,\text{in}} \exp\left(-j\frac{2\omega N_{\text{glass}}\cos\left(\Theta_{\text{glass}}\right)}{c} L_{\text{glass}}\right) \\
&\quad \left[\left(1-R_{\text{glass,air}}\right)\chi^{\text{glass}}_{s,xxz}\sin\left(2\theta_{\text{glass}}\right)\cos\left(\Theta_{\text{glass}}\right) + \left(1+R_{\text{glass,air}}\right)\chi^{\text{glass}}_{s,zxx}\sin\left(\Theta_{\text{glass}}\right)\cos^2\left(\theta_{\text{glass}}\right) + \right. \\
&\quad \left.\left(1+R_{\text{glass,air}}\right)\chi^{\text{glass}}_{s,zzz}\sin^2\left(\theta_{\text{glass}}\right)\sin\left(\Theta_{\text{glass}}\right)\right] \\
&\quad + j\frac{\omega}{2cN_{\text{glass}}\cos\left(\Theta_{\text{glass}}\right)} t^2_{\text{air,glass}} T_{\text{glass,air}} E^2_{\omega,\text{in}} \exp\left(-2j\frac{\omega n_{\text{glass}}\cos\left(\theta_{\text{glass}}\right)}{c} L_{\text{glass}}\right) \\
&\quad \left[\left(1-r^2_{\text{glass,air}}\right)\chi^{\text{glass}}_{s,xxz}\sin\left(2\theta_{\text{glass}}\right)\cos\left(\Theta_{\text{glass}}\right) + \left(1-r_{\text{glass,air}}\right)^2\chi^{\text{glass}}_{s,zxx}\sin\left(\Theta_{\text{glass}}\right)\cos^2\left(\theta_{\text{glass}}\right) + \right. \\
&\quad \left.\left(1+r_{\text{glass,air}}\right)^2\chi^{\text{glass}}_{s,zzz}\sin^2\left(\theta_{\text{glass}}\right)\sin\left(\Theta_{\text{glass}}\right)\right].
\end{aligned} \tag{3}
$$

$R_{i,j}$ and $r_{i,j}$ are the Fresnel reflection coefficients (for p-polarized light) propagating from medium i to j.

To include temporal walk-off effects we model the laser light as 100 fs (FWHM) sech^2 pulses

$$
P_{2\omega} = K_2 \int_{-\infty}^{+\infty} \left| E_{2\omega,\text{front}}\,\text{sech}^2\left(\frac{t}{\frac{\Delta t}{2\ln(1+\sqrt{2})}}\right) + E_{2\omega,\text{back}}\,\text{sech}^2\left(\frac{t+t_{\text{walk-off}}}{\frac{\Delta t}{2\ln(1+\sqrt{2})}}\right)\right|^2 \mathrm{d}t, \tag{4}
$$

with Δt the FWHM pulse duration and $t_{\text{walk-off}}$ the walk-off time.

Supplementary Figure S4: Comparison of different theoretical models for the ABC-type thin film measurements

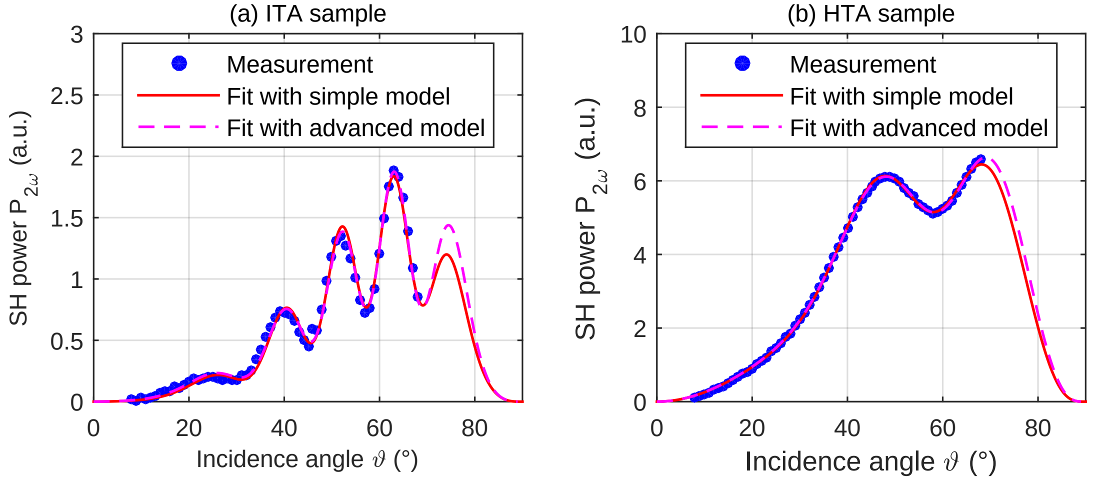

Supplementary Fig. S4 (a) shows the measurement and fitting for the ITA sample. The full red line is obtained using the relatively simple model described in the main paper. The dashed purple line is the result of fitting with a more advanced model that takes into account the birefringence of the nonlinear film, the multiple reflections inside the nonlinear thin film and the single reflections in the substrate, the finite thickness of the thin film and the temporal walk-off in the substrate. The thin film contribution is modelled according to the transfer matrix formalism described in Ref. 5. The matrix formalism is implemented in MATLAB. Though correct and very complete, the matrix formalism is not very transparent and prone to coding errors. For the contribution of the back glass-air interface we used the second term of Eq. 3. In the fitting we assume that $\chi^{\text{ABC}}_{xxz} = \chi^{\text{ABC}}_{zxx} = A^{\text{ABC}}_{zx}/3$. This is necessary even for the advanced model. If we do not use this approximation, the

relative uncertainties on the off-diagonal components can go above 100% and the results become essentially meaningless. By eliminating one fitting parameter, we can significantly reduce the uncertainty on the final results.

The fitting results are (with standard errors): $A_{zx}^{\mathrm{ABC}} = (0.211 \pm 0.011)\,\mathrm{pm/V}$, $\chi_{zzz}^{\mathrm{ABC}} = (0.974 \pm 0.054)\,\mathrm{pm/V}$ and $L_{\mathrm{glass}} = (498.26 \pm 0.16)\,\mu\mathrm{m}$, for the simple model and $A_{zx}^{\mathrm{ABC}} = (0.2251 \pm 0.0087)\,\mathrm{pm/V}$, $\chi_{zzz}^{\mathrm{ABC}} = (0.800 \pm 0.040)\,\mathrm{pm/V}$ and $L_{\mathrm{glass}} = (498.24 \pm 0.14)\,\mu\mathrm{m}$, for the more advanced model.

The same procedure is repeated for the HTA sample (see Supplementary Fig. S4 (b)).

The fitting results are (with standard errors): $A_{zx}^{\mathrm{ABC}} = (0.6045 \pm 0.0081)\,\mathrm{pm/V}$, $\chi_{zzz}^{\mathrm{ABC}} = (0.6970 \pm 0.0098)\,\mathrm{pm/V}$ and $L_{\mathrm{glass}} = (170.310 \pm 0.040)\,\mu\mathrm{m}$, for the simple model and $A_{zx}^{\mathrm{ABC}} = (0.5965 \pm 0.0046)\,\mathrm{pm/V}$, $\chi_{zzz}^{\mathrm{ABC}} = (0.634\,63 \pm 0.005\,90)\,\mathrm{pm/V}$ and $L_{\mathrm{glass}} = (169.968 \pm 0.023)\,\mu\mathrm{m}$, for the more advanced model.

The advanced model yields slightly different results, but the discrepancy is within the error margins (standard error and systematic error due to angular uncertainty).

Supplementary Figure S5: Interference fringes of s-polarized SH for $\varphi = 45°$ in KIT setup

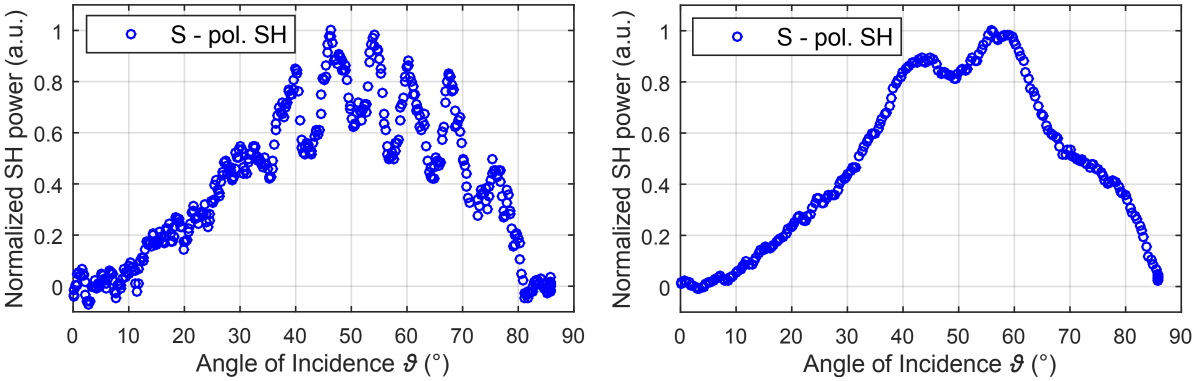

(a) Measurement on ITA sample

(b) Measurement on HTA sample

Supplementary Fig. S5 displays the normalized s-polarized SH power as a function of the angle of incidence ϑ for fixed pump polarization $\varphi = 45°$ obtained from (a) the ITA sample at and (b) the HTA sample, respectively. For this measurement configuration only the elements $\chi_{xxz}^{\mathrm{ABC}}$ and $\chi_{s,xxz}^{\mathrm{glass}}$ contribute to the SH power. The very pronounced interference fringes for the ITA sample can explain why the determined values of χ_{xxz} vary for varying angle of incidence in Table 2 of the main text. On the other hand, there are only minor interference fringes for the HTA sample, which results in minor changes in the determined values of χ_{xxz} for the specific angles of incidence.

Supplementary Figure S6: Interference fringes of p-polarized SH for $\varphi = 0°$ in KIT setup

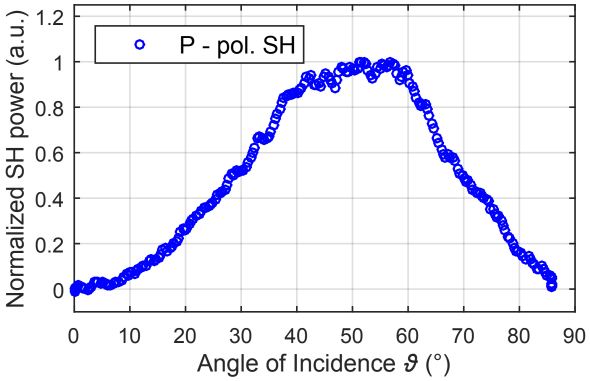

Supplementary Fig. S6 shows the normalized p-polarized SH power as a function of the angle of incidence ϑ for fixed pump polarization $\varphi = 0°$ obtained from the HTA sample. For this measurement configuration only the elements $\chi_{zxx}^{\mathrm{ABC}}$ and $\chi_{s,zxx}^{\mathrm{glass}}$

contribute to the SH power. There are no interference fringes visible for the HTA sample. Therefore it can be concluded that $\chi^{\text{glass}}_{s,zxx}$ is much smaller than χ^{ABC}_{zxx}. This finding explains why the determined values for χ^{ABC}_{zxx} for the HTA sample do not vary depending on the angle of incidence, as listed in Table 2 in the main text. Unfortunately, a corresponding measurement on the ITA sample could not be performed since the level of generated SH power is too close to the detection limit of the setup.

Supplementary Table S1: ALD deposition parameters for the HTA sample

Precursor for	Aluminium	Titanium	Hafnium	Oxygen
Temperature (°C)	not heated	80	75	not heated
Pulse duration (s)	0.015	0.1	0.15	0.015
Wait time after pulse (s)	20	see below	20	20

Parameters used for ALD. The vacuum valve was closed before the insertion of the titanium precursor and reopened two seconds later; subsequently the chamber was flushed for 20 s with 100 sccm of argon.

References

1. Dmitriev, V. G., Gurzadyan, G. G. & Nikogosyan, D. N. *Handbook of Nonlinear Optical Crystals* (Springer, Berlin; New York, 1999), 3rd edn.
2. Jerphagnon, J. & Kurtz, S. K. Maker fringes: a detailed comparison of theory and experiment for isotropic and uniaxial crystals. *J. Appl. Phys.* **41**, 1667–1681 (1970).
3. Sipe, J. E. New Green-function formalism for surface optics. *J. Opt. Soc. Am. B* **4**, 481–489 (1987).
4. Heinz, T. F. Second-order nonlinear optical effects at surfaces and interfaces. In Ponath, H. E. & Stegeman, G. I. (eds.) *Nonlinear Surface Electromagnetic Phenomena*, 353–416 (Elsevier, 1991).
5. Bethune, D. S. Optical harmonic generation and mixing in multilayer media: extension of optical transfer matrix approach to include anisotropic materials. *J. Opt. Soc. Am. B* **8**, 367–373 (1991).
